# Polymeric hydrogels for burn wound care: Advanced skin wound dressings and regenerative templates

**DOI:** 10.4103/2321-3868.143616

**Published:** 2014-10-25

**Authors:** Marta Madaghiele, Christian Demitri, Alessandro Sannino, Luigi Ambrosio

**Affiliations:** 1 Department of Engineering for Innovation, University of Salento, Via per Monteroni, 73100 Lecce, Italy; 2 Department of Chemicals Science and Materials Technology, National Research Council of Italy, Rome, Italy

**Keywords:** Burns, hydrogels, skin regeneration, wound healing, wound dressing

## Abstract

Wound closure represents a primary goal in the treatment of very deep and/or large wounds, for which the mortality rate is particularly high. However, the spontaneous healing of adult skin eventually results in the formation of epithelialized scar and scar contracture (repair), which might distort the tissues and cause lifelong deformities and disabilities. This clinical evidence suggests that wound closure attained by means of skin regeneration, instead of repair, should be the true goal of burn wound management. The traditional concept of temporary wound dressings, able to stimulate skin healing by repair, is thus being increasingly replaced by the idea of temporary scaffolds, or regenerative templates, able to promote healing by regeneration. As wound dressings, polymeric hydrogels provide an ideal moisture environment for healing while protecting the wound, with the additional advantage of being comfortable to the patient, due to their cooling effect and non-adhesiveness to the wound tissue. More importantly, recent advances in regenerative medicine demonstrate that bioactive hydrogels can be properly designed to induce at least partial skin regeneration *in vivo*. The aim of this review is to provide a concise insight on the key properties of hydrogels for skin healing and regeneration, particularly highlighting the emerging role of hydrogels as next generation skin substitutes for the treatment of full-thickness burns.

## Introduction

According to the World Health Organization, more than 300,000 deaths occur each year as a consequence of fire-induced burns, with additional deaths ascribed to scalds and other forms of burns (*e.g.* caused by electricity, chemicals, radiation, *etc*.)[1] Burn injuries are indeed among the most challenging ones to manage. Significant fluid loss and extensive tissue damage, resulting from deep wounds, impair multiple vital functions performed by skin.[2] Wound infection, which further increases the local tissue damage, is a common complication, while systemic inflammatory and immunological responses might lead to a higher predisposition to life-threatening sepsis and multi-organ failure.[2–4] In such cases, early and appropriate clinical treatments are fundamental to reduce the mortality rates associated to the injury.[5]

**Table:** 

Access this article online	
**Quick Response Code**:	**Website**: www.burnstrauma.com
	**DOI**: 10.4103/2321-3868.143616

Wound closure is a paramount target to achieve, especially in the treatment of deep and/or extensive burns, where the dermis layer is partially or totally destroyed and the intrinsic capability of spontaneous re-epithelialization is greatly reduced or absent.[5–7] However, even when wound closure is attained, the clinical outcome is often far from being optimal. The physiological healing or repair of deep injuries indeed involves contraction and formation of an epithelialized scar, which cause esthetic and functional impairment.[5–8] In most cases of massive injuries, the impact of scar tissues on the body is devastating, and later plastic surgery procedures can only attempt to reduce the deformity and/or ameliorate the appearance of the tissues.[8–10] In this context, induced skin regeneration, which is opposed to repair as an endpoint of healing, appears as the only way to go to truly improve the quality of life of burned patients.

This review focuses on the use of polymeric hydrogels either as wound dressings or as regenerative templates, which are meant to promote wound closure and/or skin regeneration following burn injuries. In particular, the intrinsic healing potential of hydrogels is discussed in the following, in comparison with different strategies and biomaterials adopted for burn wound care. Along with the classical use of hydrogels as primary wound dressings, some advanced experimental scenarios are presented, where hydrogels show promise as effective ‘regenerative templates’ capable of promoting skin regeneration.

## Current and prospective use of polymeric hydrogels in burn wound care

### Hydrogels as wound dressings for superficial and partial-thickness burns

**Figure 1:**
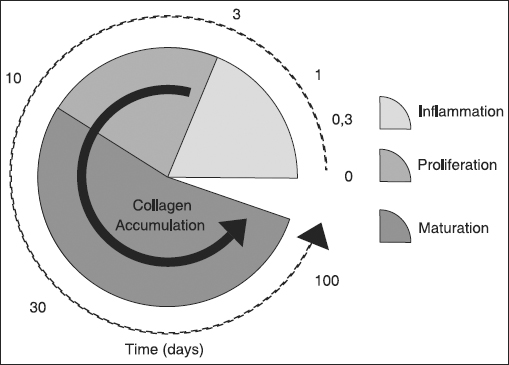
Timing and phases of skin wound healing. Following the inflammatory response, a disorganized and loose collagen matrix (granulation tissue) is formed and epithelialized during the proliferation phase. Final remodeling or maturation takes place over several weeks, when the collagen matrix is gradually reorganized by myofibroblasts along lines of tension, which arise from wound contraction. The final outcome of healing can thus be regarded as the sum of two processes, that is contraction and formation of epithelialized scar.

Following a burn injury, the wound healing process [Figure 1], as well as the time required for healing, will basically depend on the thickness of the injured dermis layer [Table 1]. Prompt and appropriate burn care appears crucial for optimal healing and final appearance of the scar, since burn depth might dangerously increase if the wound dries or become infected[11] and scar synthesis and contracture typically worsen for delayed wound closure. As comprehensively reviewed in the literature,[12] a number of specialized primary wound dressings are currently available for the treatment of partial-thickness burns (both superficial and deep partial-thickness burns) and other types of wounds, with low to high levels of wound exudates. Such dressings are designed to absorb the wound exudate, thus keeping a moist environment to facilitate debridement of the necrotic tissue and spontaneous re-epithelialization of the skin,[13] while providing temporary and prompt wound coverage and a mechanical barrier to infections. Several dressings might also include specific antibiotics or different antibacterial agents (*e.g.* silver ions) in their formulation, in order to further protect the wound bed from undesired microbial contaminations.

**Table 1:** **Burn classification and healing response**

Burn classification	Depth	Wound appearance	Spontaneous wound closure (Yes/No)
1^st^ degree			
Superficial	Epidermis	Red, dry, painful, turns white when pressed	Yes; epidermal regeneration
2^nd^ degree			
Superficial partial-thickness	Papillary (superficial) dermis	Red, moist, blistering, painful, turns white when pressed	Yes; epithelialization with no or negligible scar
Deep partial-thickness	Reticular (deep) dermis	Red and white, blistering, painful	Yes (only if not large); epithelialization and scar formation
3^rd^ degree			
Full-thickness	Dermis and subdermal tissues	White, leathery or charred, dry, no pain	No

**Figure 2:**
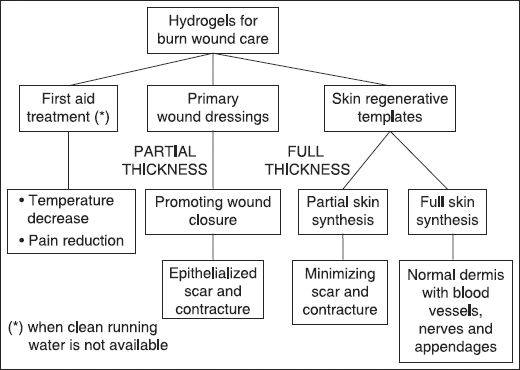
Synoptic scheme of the current and prospective use of hydrogels in burn wound care.

Due to their hydrophilic nature and soft tissue-like properties, polymeric hydrogels emerge over different types of biomaterials as prime candidates for the development of wound dressings for the treatment of burns and other skin lesions [Figure 2].[14,15] Hydrogels are macromolecular networks, stabilized by means of chemical or physical interactions among the polymer chains, which are able to retain large amounts of water in their mesh structure. The dissolution of the polymer in the solvent is indeed prevented by the crosslinking nodes existing among the macromolecules. Due to this peculiar structure, hydrogels show a hybrid behavior, with mechanical (elastic) properties similar to those of a solid, but diffusive properties matching those of a liquid. Remarkably, hydrogels can absorb and release water in a reversible manner, in response to specific environmental stimuli, *e.g.* temperature, pH and ionic strength.[16,17] Such a smart behavior towards changes of physiological variables suggests their use in a variety of biomedical applications.[16–23]

The intrinsic potential of hydrogels to promote skin healing has been increasingly investigated and applied in the clinical setting since the early eighties. First of all, hydrogels absorb and retain the wound exudate, thus promoting fibroblast proliferation and keratinocyte migration, which are both necessary for complete epithelialization of the wound.[13,24,25] Furthermore, the tight mesh size of hydrogels (in the order of 100 nm in the swollen state) prevents bacteria from reaching the wound,[17] while still allowing for an efficient transport of bioactive molecules (*e.g.* antimicrobial agents and pharmaceuticals) to the wound bed. Such molecules can be easily entrapped in the polymer network during the gelling process, in order to be gradually released to the wound as the hydrogel absorbs the exudate and swells.[26–31] The peculiar and tunable mechanical properties of hydrogels also provide them with suitable elasticity and flexibility to adapt to wounds located in different body sites.

Wearing comfort and immediate pain relief are likely the most advantageous features of hydrogels to the patients, if compared to traditional bandages, pads or gauzes. In case of burns, if clean running water is not available for first aid, application of hydrogels onto the wound is the only way to cool the wound, in order to minimize the extent of damage and reduce pain.[32,33] The high water content of hydrogels makes them particularly cooling and soothing on the wounded area. Hydrogels are also non-adhesive, since cells do not readily attach to highly hydrophilic surfaces. This implies that hydrogel dressings do not stick to the wound, highly facilitating the change of the dressing by causing less pain and discomfort to the patient. Hydrogel transparency, which may depend on the crosslink density of the polymer network, is an additional advantage over traditional bandages, as wound healing can be constantly monitored without removing the primary dressing.

An enormous range of hydrogel dressings is commercially available for the treatment of minor burns and other skin wounds, in the multiple forms of amorphous gels, gel-impregnated gauzes, sheets or plasters.[13,26,34] While amorphous gels are preferred for cavity wounds, sheets and gel-impregnated gauzes find application mainly in the treatment of superficial burns.[13] Plaster-like hydrogel dressings (*e.g.* MySkin®) are particularly attractive for their easy use and removal, as they can be correctly positioned onto the wound without the need for additional dressings (adhesives and bandages).

**Figure 3:**
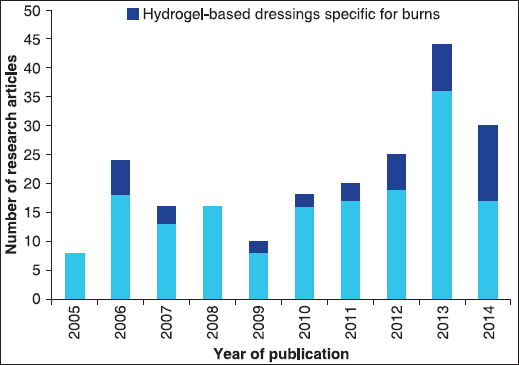
Increasing number of research articles concerning the development of hydrogel-based wound dressings, published in the last ten years (Source: PubMed. Keywords: hydrogel wound dressing; burn. Date: September 12, 2014).

In spite of the various hydrogel-based products already on the market, the development or optimization of advanced hydrogel dressings still represents a very active research field, with the aim of further improving skin healing in relation to specific clinical applications [Figure 3]. In particular, there is a growing tendency in the development of hydrogel formulations that encompass multiple materials [Table 2], in an attempt to simultaneously address different aspects of wound healing (epithelialization, collagen synthesis, vascularization, contraction) and wound management (*e.g.* infection control, dressing flexibility).[35–52] *In situ* forming gels[38,53–56] and radiation-crosslinked gels[36,44,45,57] are also appealing for the development of novel wound dressings.

**Table 2:** **A few examples of hydrogel-based dressings and regenerative templates investigated in recent literature for the treatment of partial- and full-thickness burns, respectively**

Burn depth	Hydrogel precursor(s)	Additional component(s)	Reference(s)
Partial-thickness	AMPS/PEGDA	—	[20]
	AMPS	Silver nanoparticles	[27],[36],[37]
	Chitin	ZnO nanoparticles	[29],[30]
	Chitosan	—	[15],[38]
	Keratin	—	[39]
	Laponite®/alginate (*)	Mafenide	[40]
	PVA/chitosan	Silver sulfadiazine	[28],[41]
	PVA	Silver nanoparticles	[42]
	PVA/lysine/vanillin	—	[43]
	PVP/PEG	Honey	[44]
	PVP/PEG	Sea cucumber	[45]
	Self-assembling peptides	—	[46],[47]
Full-thickness	Chitosan	—	[25]
	Chitosan/collagen	Lysostaphin	[31]
	Collagen/PEG/fibrin	—	[48]
	Dextran/PEGDA	—	[49]
	Dextran	Chitosan microparticles with EGF and VEGF	[50]
	Hyaluronan	—	[51]
	Hyaluronan/gelatin/PEGDA (Extracel®)	—	[52]

AMPS = 2-acrylamido-2-methylpropane sulfonic acid sodium salt, PEGDA = polyethylene glycol diacrylate, PVA = Polyvinyl alcohol, PVP = Polyvinyl pyrrolidone, PEG = polyethylene glycol, EGF = epidermal growth factor, VEGF = vascular endothelial growth factor, (*) Laponite is a gel forming clay

## Hydrogels as regenerative templates for full-thickness and extensive burns

### Skin substitutes in the clinical practice

In all cases where spontaneous and effective epithelialization does not occur, such as for full-thickness and/or extensive burns, wound closure requires surgical intervention, by means of excision of burned skin and subsequent grafting.[7] Autograft is still the best option for skin replacement, but is practically challenged, in some cases, by the unavailability of donor sites and the critical conditions of the patient. Although the Meek technique[58] can be applied to enlarge the effective surface area covered by autologous split thickness skin grafts, the yielded expansion ratio (1:9) might still be too low for the treatment of extensive burns. Other limitations of autograft deal with the co-morbidity of the harvesting sites, resulting in additional scarring.[7] Several skin substitutes, either complementary or alternative to autografts, have thus been developed in order to close the wound, promote healing and possibly replace the missing skin.[7,9,58–61]

Temporary substitutes are applied to excised wounds until complete healing is achieved or further grafting is performed. In addition to natural grafts (*e.g.* human cadaver allografts, human amnion and xenografts), synthetic (*e.g.* Biobrane®) and bioengineered (*e.g.* TransCyte®) membranes, based on biocompatible and non-degradable polymers (*e.g.* nylon, silicone), are adopted as temporary coverage of excised wounds.[60–62] Conversely, permanent skin substitutes are designed to indefinitely replace one or more skin layers, by delivering exogenous cells and/or degradable materials, the latter being able to stimulate healing and undergo remodeling *in vivo*. It is in this context that skin substitution eventually meets skin regeneration.

Epicel® or cultured epidermal autograft (CEA) is currently available for replacing the single epidermal layer in cases of large burns (covering greater than 30% total body surface area).[60] However, its mechanical fragility, due to the absence of supportive dermis, is a major drawback and limits its use. Regeneration of the dermis, which sustains irreversible injury (that is wound closure attained by means of contraction and formation of scar tissue), is the truly challenging target in skin regeneration.[6] Suitable biomaterial-based ‘regenerative templates’, also termed scaffolds, play a pivotal role in instructing endogenous cells to the synthesis of new tissue that mimics, as closely as possible, the structure of undamaged dermis. The design of such regenerative templates is particularly focused on identifying and delivering structural, chemical and/or biological cues that can effectively recapitulate the complexity of the physiological dermal microenvironment, in order to steer cells towards tissue regeneration. In particular, there is growing evidence that skin regeneration can be at least partially induced by a suitable three-dimensional template able to block or reduce myofibroblast-mediated contraction, from which scar tissue seems to arise,[6] and promote a fast angiogenesis.[49] Vasculature is particularly important in large wounds, where newly formed blood vessels provide adequate nutrition and oxygen supply to the growing tissues.[63,64] A proper restoration of blood vessels, synchronized with the granulation tissue formation, is known to determine whether partial-thickness burns heal promptly or degenerate into full-thickness ones.

Several regenerative templates are currently available in the clinical practice, as reviewed in recent literature, although none of them has been reported so far to induce full skin regeneration.[7,59–62] Regenerated skin indeed lacks dermal appendages (*e.g.* hair follicles, glands)[6] and proper re-innervation.[65] Integra® DRT (Dermal Regeneration Template) is the most widely used, cell-free scaffold for the treatment of deep burns. It is composed of a collagenglycosaminoglycan sponge, possessing an equiaxed porous structure, and a silicone membrane on one side, that provides the epidermal barrier function. Once implanted, the scaffold hosts the regeneration of a functional dermis, in a period of approximately 3–6 weeks. The silicone layer is finally removed and replaced by an epidermal graft (*e.g.* autograft, Epicel®). Experimental evidence show that the main mechanism by which Integra® scaffolds induce partial skin regeneration is the effective inhibition of myofibroblast-mediated contraction.[6] The surface area of the scaffold available for cell attachment (the density of ligands for myofibroblasts) and its equiaxed porosity are thus the key variables affecting the bioactivity, in addition to its degradation rate.[6]

Unlike Integra®, Apligraf® is a bi-layered product for the treatment of burns that contains viable cells. The surface layer is formed by neonatal keratinocytes, while the inner layer is made up of neonatal allogeneic fibroblasts dispersed into a collagen hydrogel. The use of exogenous keratinocytes allows the *in vitro* formation of a cornified epidermis, so that the resulting product can close the wound bed, once grafted. Several clinical studies demonstrate that Apligraf® is able to expedite the healing of full-thickness burns and other wound types, while limiting scar formation and favoring partial skin regeneration.[60] The exact mechanism of action of Apligraf® is not known, but exogenous fibroblasts are likely to accelerate the synthesis of the extra cellular matrix (ECM) and be an invaluable source of cytokines and growth factors that are important for wound healing. Whether and how the collagen gel itself plays a specific role in guiding tissue regeneration, in addition to simply act as a delivery vehicle for exogenous cells, has not been elucidated.

### Potential of hydrogels for induced skin regeneration

Many research efforts are currently directed to the development and optimization of regenerative templates, in order to promote complete skin regeneration. In this scenario, the traditional view of hydrogels as temporary wound dressings is being replaced by the idea of hydrogel-based regenerative templates, or permanent skin substitutes [Figure 2 and Table 2].[14,52,66–69]

A detailed analysis of the number of hydrogels so far proposed for skin regeneration would be overwhelming and go beyond the purposes of the present review. In the following, the potential of hydrogels for inducing skin regeneration is discussed, with a few hints to significant findings reported in recent literature.

In general, hydrogels display several properties that make them attractive for tissue engineering. Most hydrogels (especially self-assembling ones) exhibit a structure similar to the ECM[46] and rubbery mechanical properties compatible with those of soft tissues, which suggest their possible role as instructive and permissive scaffolds for many tissue types.[70] It is well-known that the three-dimensional network-like matrix provided by hydrogels induce embedded cells to behave differently with respect to cells seeded on two-dimensional substrates, *e.g.* in terms of migration[71] and ECM deposition.[72] Moreover, since the gelling process is usually biocompatible,[16,17] hydrogels can be exploited to deliver different active molecules to the site of interest, such as cytokines, growth factors and antibiotics,[56,69,73] as well as viable exogenous cells, *e.g.* epithelial cells, fibroblasts and stem cells.[18,48,74–76] The chemistry of hydrogels can also be easily modified with ECM domains and/or selected functional groups, in order to promote both cell adhesion (which otherwise would be very low, as mentioned above) and specific cell functions.[49,77]

Looking at the particular case of skin regeneration, the specific mechanisms whereby a hydrogel matrix may instruct skin cells towards regeneration, rather than repair, represent an exciting field of investigation and are not fully understood yet. A recent study has attempted to detail the biological events occurring at the edges of a full-thickness wound, following implantation of different scaffolds.[78] Remarkably, while porous collagen and alginate scaffolds were found to impede re-epithelialization and increase the inflammatory response, alginate hydrogels did not elicit similar responses and appeared to be much more biocompatible. However, the specific hydrogel-tissue interactions that may lead to tissue regeneration have not been addressed.

Hydrogel bioactivity *in vivo* clearly depends on microstructural parameters (*e.g.* chemical composition, crosslink density, mesh size), which may directly affect cell behavior, and macroscopic properties (*e.g.* mechanical stiffness, degradation rate), which may indirectly affect cells and may be important for integration and remodeling in the host tissue.

Provided that cell adhesion is preliminarily enhanced to facilitate cell-biomaterial interactions, the mechanism by which cells infiltrate and migrate through the intricate polymer matrix is likely related to its degradation (physical infiltration of cells is indeed inhibited by the narrow size of the polymer mesh).[71,79] Hydrogel degradation usually takes place by means of hydrolysis and/or enzymatic digestion, the latter activated by cells themselves. Interestingly, recent studies on chitosan- and dextran-based hydrogels show that infiltration of inflammatory cells (*e.g.* neutrophils) into the matrix can significantly accelerate the degradation process, thus suggesting a close interrelation between hydrogel degradation and cellular infiltration.[18,49] A rapid degradation of the polymer matrix by macrophages has also been reported for hyaluronic acid-based hydrogels.[51] In general, the degradation rate of the scaffold should match the rate of tissue formation, or at least be comprised within an optimal range, since a too slow degradation would interfere with remodeling and a too fast degradation would lead to premature scaffold resorption. For the healing of full-thickness burns in a mouse model, rapid degradation of dextran-based hydrogels, taking place over 7 days, was found to be optimal to promote a prompt migration of angiogenic cells and neovascularization of tissues.[49] Remarkably, by day 21, burn wounds treated with the hydrogel were able to develop a mature epithelial structure with hair follicles and sebaceous glands, while new hair growth was detected after 5 weeks of treatment.[49] Such findings are particularly outstanding and exciting, if compared with the partial skin synthesis (skin with no appendages) achieved by currently available skin substitutes. Although the specific regenerative mechanisms elicited by the dextran-based hydrogel deserve further investigation, the obtained results showed that the hydrogel degradation rate played a key role in regulating the process of neovascularization, which in turn may affect the quality of dermal regeneration. Remarkably, a hydrogel alone, free of exogenous cells and cytokines, was used. The application of a cell-free scaffold (like the successful Integra®) is clearly attractive for a translational medicine approach, where a fast transition from the lab bench to the hospital is desired.

However, alternative and more complex approaches to full skin regeneration, where the scaffold delivers exogenous cells, growth factors and various cytokines, are also under investigation and pave the way for future clinical scenarios. Bio-printing techniques hold promise to produce hydrogels with controlled spatial distribution of cells and/or bioactive molecules, which might also work as valuable platforms for the analysis of the mechanisms underlying cell-material interactions and, more specifically, hydrogel-induced tissue regeneration.[52,72] Bio-printed hydrogels containing devices for controlled drug delivery (*e.g.* microparticles) are likely to allow for both spatial and temporal gradients of bioactive molecules within the construct.[80]

Proper re-innervation of regenerated skin is another aspect of complete skin regeneration that unfortunately is often neglected, in spite of being particularly significant for functional recovery.[65] Future investigations should therefore be directed to optimize skin regenerative strategies, with the aim of enhancing simultaneous nerve regeneration. Notably, there is a close association, even in development, of blood vessels and nerves, which suggests that angiogenic molecules, as well as hydrogels with known angiogenic potential (*e.g.* hyaluronan-based), might also play a key role in nerve regeneration.[81]

With specific regard to burn injuries, it is worth citing a recent approach to wound closure, which combines tissue engineering strategies with ‘gold standard’ autologous skin micrografts.[82] Briefly, nanofibrous scaffolds and skin micrografts are coupled in sandwiched structures, in order to achieve higher expansion ratios and promote re-epithelialization *in vivo*. The creative idea of sandwichtype transplants shows potential to be extended to different scaffold types, including hydrogels, and holds promise for future clinical application.

The unique properties displayed by hydrogels as tunable and ‘printable’ tissue-mimicking matrices make them promising biomaterials for the synthesis of next generation skin substitutes. The experimental results reported above for dextran-based hydrogels seem also to suggest that full skin regeneration may be a realistic goal in the future.

However, in spite of the exciting findings in several animal studies, it is worth noting that ongoing clinical trials on burn treatment are mostly focused on post-market analyses of several hydrogel dressings (*e.g.* Aquacel® AG, MySkin® Patch, Prontosan® Wound Gel X), rather than testing the efficacy of novel skin regenerative templates.[83] It may be argued that several regulatory, financial and commercial constraints delay the development of skin substitutes, if compared to wound dressings, especially when complex tissue engineering strategies are involved.

## Conclusion

Due to their peculiar properties, hydrogel-based dressings are ideal to facilitate and accelerate wound healing, thus find large use in the treatment of partial-thickness burns. Moreover, recent findings highlight the further potential of hydrogels to induce skin regeneration in full-thickness wounds. Although future studies are necessary to elucidate the regenerative mechanisms activated by hydrogels, the combination of hydrogels with advanced tissue engineering strategies holds promise for enhanced skin regeneration in the clinical setting. To this aim, regulatory and cost-effective aspects should be properly addressed in the design of novel skin substitutes.

## Author Help: Online Submission of the Manuscripts

All manuscripts must be submitted on-line through the website http://www.journalonweb.com/bt. First time users will have to register at this site. Registration is free but mandatory. Registered authors can keep track of their articles after logging into the site using their user name and password. Generally, the manuscript should be submitted in the form of two separate files. Images should be submitted sepatately.

### (1) Title Page/First Page File/Covering Letter

Prepare the title page, covering letter, acknowledgement etc. using a word processor program. All information related to your identity should be included here. Use text/doc/pdf file. Do not zip the files.

### (2) Blinded Article File

The main text of the article, beginning from Abstract till References (including tables) should be in this file. The file must not contain any mention of the authors’ names or initials or the institution at which the study was done or acknowledgements. Page headers/running title can include the title but not the authors’ names. Use rtf/doc files. Do not zip the files. **Limit the file size to 1 MB**. Do not incorporate images in the file. If file size is large, graphs can be submitted as images separately without incorporating them in the article file to reduce the size of the file. The pages should be numbered consecutively, beginning with the first page of the blinded article file.

### (3) Images

Submit good quality color images. **Each image should be less than 2 MB in size**. Size of the image can be reduced by decreasing the actual height and width of the images (keep up to 1600 × 1200 pixels or 5–6 inches). Images can be submitted as jpeg files. Do not zip the files. Figures should be well labeled and provided with brief but informative figure legends, being self-explanatory. Legends for the figures/images should be included at the end of the article file.
